# Exploring the Roles and Responsibilities of an Educational Supervisor in Medical Training Within the United Kingdom

**DOI:** 10.7759/cureus.41244

**Published:** 2023-07-01

**Authors:** Umar N Said, Khalid Al-Hashimi, Taherah N Khan, Omar M Ismail, Omar El-Omar

**Affiliations:** 1 Trauma & Orthopaedics, University Hospitals Coventry & Warwickshire, Coventry, GBR; 2 Vascular Surgery, Colchester General Hospital, Colchester, GBR; 3 General Medicine, Worcestershire Acute Hospitals NHS Trust, Worcestershire, GBR; 4 Trauma & Orthopaedics, Royal Lancaster Infirmary, Lancaster, GBR; 5 Trauma and Orthopaedics, King’s Mill Hospital, Nottinghamshire, GBR

**Keywords:** supervision, supervisor, educational, uk, education, medical

## Abstract

Medical trainees or junior doctors within the United Kingdom, regardless of their level of training or specialty, are assigned an educational supervisor (ES). The General Medical Council within the United Kingdom defines an ES as “a trainer who is selected and appropriately trained to be responsible for the overall supervision and management of a specified trainee’s educational progress during a clinical placement or series of placements.” This article critically evaluates the current literature to explore the roles of the ES in supporting and monitoring a trainee’s progress while discussing challenges associated with the role. Through clearer delineation of the role of an ES, barriers to improving training can be identified and overcome, thus improving overall satisfaction with training.

## Introduction and background

To understand the role of an educational supervisor (ES) we must first look at the way in which postgraduate medical education is supervised. Within postgraduate medical education, there has been a degree of supervision for trainees; however, this tended to be in the form of one supervisor who encompassed the roles of both a clinical supervisor (CS) and an ES. During the implementation of Modernising Medical Careers (MMC) in 2005, there was clearer differentiation between the roles of a CS and an ES with individual roles and responsibilities defined for each [1].

The clearly delineated roles of a CS and an ES within the postgraduate medical education setting are in their relative infancy, as mentioned above. An ES has been defined as “a trainer who is selected and appropriately trained to be responsible for the overall supervision and management of a trainee’s educational progress during a series of placements” [2]⁠. A CS has been defined as “A trainer who is selected and appropriately trained to be responsible for overseeing a specified trainee’s clinical work and providing constructive feedback during a training placement” [3]. Though standardised definitions have been placed in attempts to clearly outline variations between the two roles, crossover naturally occurs in the supervision of clinical juniors and monitoring their clinical development. This article intends to appraise the current literature to critically evaluate the roles and responsibilities of the ES in gauging and supporting a junior’s progress while also addressing challenges associated with the role. The provision of more clear direction in the supervision of trainees subsequently results in more targeted approaches in mitigating weaker areas of performance and overall improving trainee and ES satisfaction.

## Review

To ensure that an ES is performing their role correctly, they need to have been provided with adequate training. Since the implementation of MMC, the role of an ES has become a requirement within postgraduate medical education to support a trainee’s progression throughout their desired speciality or career pathway. There are several essential training modules that an ES must attend which are highlighted in the ES handbook, as published by Health Education England [4]. These essential training courses include a mentor study day, an associated workbook, a mentoring guide, and, ideally, but not as a requirement, the ES may have or be working towards a postgraduate certificate in education.

The General Medical Council (GMC) has also adopted seven main criteria by which an ES must be able to provide evidence to ensure they are performing the role of an ES appropriately. These seven criteria are highlighted and were derived from The Association of Medical Educators guidance [5]. Ensuring safe and effective patient care through training, establishing and maintaining an environment for learning, teaching and facilitating learning, enhancing learning through assessment, supporting and monitoring educational progress, guiding personal and professional development, and continuing professional development as an educator.

If we consider what has been discussed above from the point of view of an ES, we can begin to appreciate some of the challenges that may be experienced in trying to achieve what is expected of an ES. Through a survey carried out by medical trainees, it was apparent that the duration of meetings with their ES only lasted an average of 10-20 minutes [6]⁠. Some potential reasons for meetings lasting such a short amount of time could include consultants not being aware of the importance of educational supervision to their trainees as mentioned by [7]⁠.

It is also important to keep in mind that when a consultant decides to become an ES they are given an extra 0.25 programmed activities per week for each trainee, which accounts for one hour per trainee they are supervising per week [4]. As mentioned above, it is also preferable that the ES is working towards some form of additional qualification in medical education; however, with no additional time allocated to perform these roles, it raises the question as to why the ES would perform an additional qualification for no real benefit to themselves. The vast majority of ES meetings only occur once per placement, and for an average of 10-20 minutes, which raises the question as to what the additional rostered time that is supposed to be used for ES is actually being used for by supervising consultants.

ES have the ability to delay a trainee’s progression throughout their career; however, there is no clear process by which they are able to be held accountable if they are performing poorly in their role as supervisors. Perhaps a way to hold ES accountable to a high standard would be to ensure that the trainees they are supervising are required to give feedback on their supervisors, and those ES who are performing poorly would then be given either additional training or removed from the role altogether.

It is important to consider how a trainee’s progress throughout a placement is assessed by their ES. The usual process involves two face-to-face meetings at the beginning and end of the placement. Some of my colleagues have also had additional meetings with their ESs in between these if they have been facing difficulties within their training or required additional support or advice. The goal of these meetings is to ensure that the trainee is adhering to the requirements of their specific training programme or placement. If a trainee is struggling in any specific area, it is the ES’s job to identify where this is and how they can then address areas in which they are struggling to meet their training requirements.

Ways in which an ES can identify or monitor an individual trainee’s progress include reviewing foundation programme assessments located within an individual’s ePortfolio [8]. Some of these assessments include direct observation in the workplace or reviewing multi-source feedback forms. Reviewing an individual’s reflective pieces, including any reflections on critical incidents or events and what they have learnt from them. Ensuring that an individual’s portfolio contains the minimally required number of case-based discussions, mini-clinical exercises, and directly observed procedural skills.

For the trainee to improve, it is vital that the ES is able to give adequate and effective feedback. Feedback is a vital tool in postgraduate medical education in terms of driving change and influencing a trainee’s learning process. The purpose of giving feedback is that it causes the trainee to learn going forward, as well as increase their self-awareness as to what needs to be learnt [9]. There is no clear pre-defined format as to how feedback should be delivered in terms of educational supervision. Research has shown that trainees are keen to receive more feedback, and the feedback they want should be adequate enough to guide their further development whether that be reflective or in terms of career development [10].

Feedback should be provided to the trainee in a timely manner. Feedback for individuals is vital as it informs an individual of their progress and the areas in which they need to improve, as well as gives them the motivation required to engage in appropriate activities in their own time [11]. Feedback should be given in a constructive manner and should not bring a trainee down as this has been shown to lower an individual’s performance. Feedback is not and should never be portrayed to the trainee as sarcasm, humiliation, criticism, or non-constructive opinions.

Some other potential barriers for an ES to give feedback to a trainee include generic factors such as lack of time within meetings, the inability of a trainee to respond to feedback that is given, or being inadequately trained in giving effective feedback [12]. Negative feedback can have a disastrous impact on a student’s progression, as identified by Shute et al., who found that negative feedback led to poor self-esteem and eventually lower student performance. However, as an ES, the issue of providing constructive feedback to a trainee with good intentions being not well received does exist [12]. Equally, from the viewpoint of an ES, it can be difficult to provide constructive feedback due to the fear of upsetting the trainee. Further barriers to giving feedback include a lack of respect for the source of feedback, defensive behaviours from trainees, generalised feedback, and physical barriers to providing feedback such as noise or a lack of protected time [13].

There are many approaches to providing feedback within postgraduate medical education,⁠ including feedback sandwich, reflective feedback conversation, agenda-led outcome-based analysis, feeding forward, and Pendleton’s rules [14]. The most commonly used within the healthcare setting when providing feedback to colleagues are Pendleton’s rules [15]. The aim of Pendleton’s rules is to engage the person receiving the feedback to self-reflect and balance positive and critical feedback. Some criticisms of offering feedback using this framework include the fact that it is a rigid mechanism and relatively formulaic; however, other feedback methods, as described above, can be used if this particular mechanism of delivering feedback is proving difficult.

Reflection is also an important tool in the educational process of junior doctors and consultants alike that helps doctors strive to be the best holistic practitioners they can be. A key responsibility of the ES is to drive the reflective process where possible and encourage the trainee to not only write reflective pieces on experiences they have had but to become involved in other activities that stimulate this process. Activities such as journal clubs, Balint groups, Schwartz groups, and quality improvement projects can all help doctors become more reflective practitioners [16].

Essentially, supervision within medical education originated from the apprenticeship model, whereby the expert (usually a consultant in a specific field) passes on their knowledge and skills to the apprentice (a more junior clinician) [17]. This naturally lends the role of an ES as a mentor or coach to their trainee, which, in turn, aids in their development. Coaching can be useful but the main roadblock to this approach is the amount of time that an ES has to spend with their trainee.

It is pivotal to understand that currently it is estimated that four generations of clinicians are working together all of whom have different learning experiences. Recent research has highlighted that trainees from different generations have different expectations in terms of supervision [18]. The most recent group of medical professionals also known as millennials expect feedback to be given in a short turnaround [19]. It has also been stated that annual or quarterly evaluations will be insufficient to meet the needs of recently graduated medical trainees. The biggest barrier to an ES in terms of providing adequate feedback and evaluations is again time, perhaps this could be solved by weekly or bi-weekly check-ins with their trainees done virtually via Microsoft Teams or Zoom. Virtual check-ins would negate the need for booking rooms and increase the flexibility of meetings.

As well as being responsible for monitoring a trainee’s progress, an ES must also be able to recognize a trainee who is in need, also known as a “trainee in difficulty.” A trainee may be in this predicament for any number of reasons, including ill health, difficulty passing professional exams, or significant life events such as the death of a close family member. There are signs that may raise concerns that a doctor is struggling or having difficulty [20]. Some of these include being difficult to contact, constantly late, and taking frequent sick leave; a rate of work which is considerably slower than their colleagues (some examples include being slow to clerk patients, arriving early, and leaving late); aggression within the workplace towards other team members; poor tolerance for criticism including being defensive; and difficulty passing professional exams and being unsure about the direction of their future.

Following the identification of a trainee in difficulty, the ES should aim to offer support and manage the situation proactively to ensure the patient’s and the trainee’s safety. Problems should be addressed as soon as possible and should not be delayed until the end of the placement. The ES should also be able to direct the trainee to appropriate services such as pastoral care in terms of personal issues, as well as have an awareness of other services including the British Medical Association and GMC where a matter of patient safety or legality is involved. Challenges to an ES performing these roles may be due to inadequate training in this subject which should be addressed in mandatory training that all ESs have to attend.

## Conclusions

The role of an ES is complex and encompasses somebody who is able to provide effective feedback, ensure a trainee is aware of what they need to do to progress and ensure they remain on track to do this, support the trainee in terms of their learning needs, and be able to recognize when a trainee is in difficulty.
